# Nonfollicular papules in the bilateral inframammary region

**DOI:** 10.1016/j.jdcr.2023.07.035

**Published:** 2023-08-09

**Authors:** Madiha Khan-Walton, Katerina Warda, Sarah Stano, Aurel Apple, Cindy Hoffman, Charles Gropper, Pooja Srivastava, Paul Chu

**Affiliations:** a New York Institute of Technology College of Osteopathic Medicine, Glen Head, New York; b Department of Dermatology, St. Barnabas Hospital, Bronx, New York; c Department of Dermatopathology, Montefiore Medical Center and Bridge Dermpath, Bronx, New York; d Department of Dermatopathology, Bridge Dermatopathology, Tarrytown, New York

**Keywords:** atypical papillary dermal elastolysis, papillary dermal elastolysis, PDE, pseudoPXE, PXE-PDE

## Background

**Fig 1:**
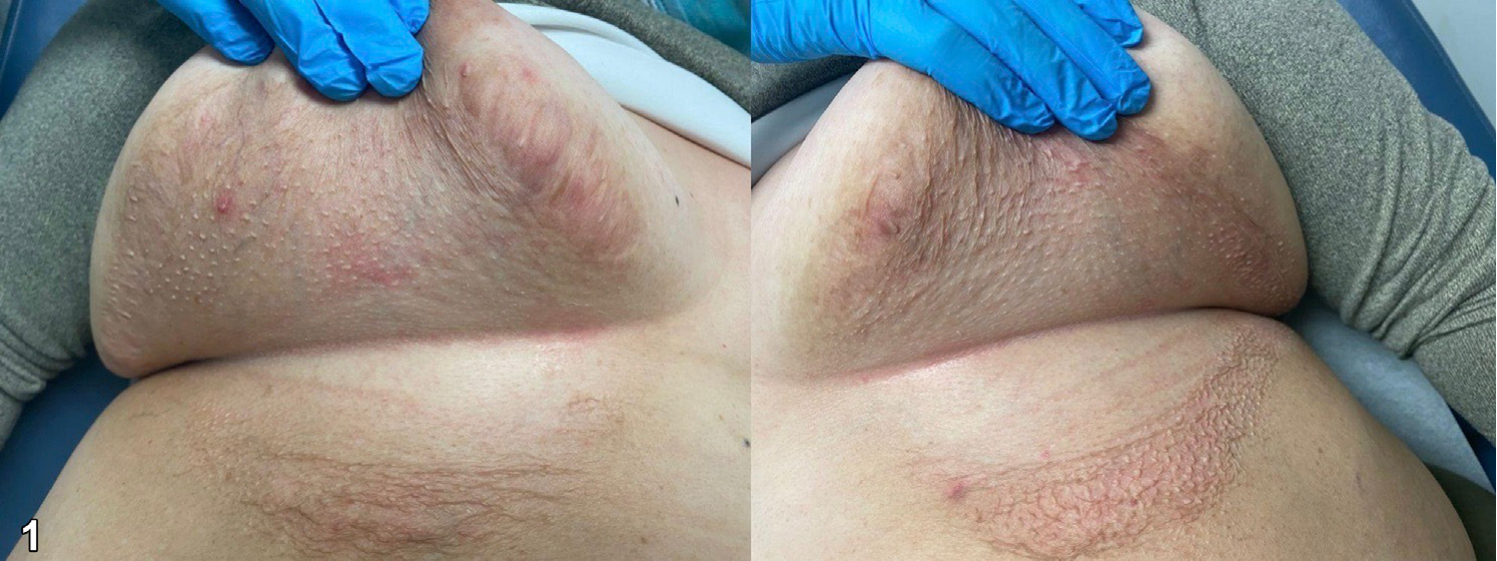

**Fig 2:**
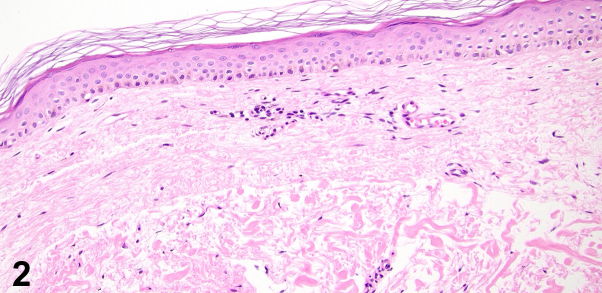

**Fig 3:**
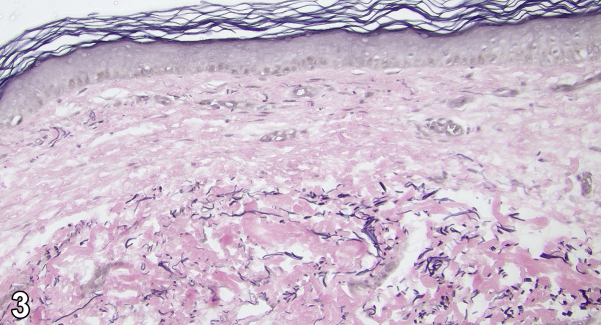

A 56-year-old female with a history of diabetes, hypertension, and hyperlipidemia presents with a 6 month history of rash under bilateral breasts for which treatment with nystatin powder and ketoconazole cream demonstrated no improvement. On examination, loose atrophic skin and white-to-yellow nonfollicular papules coalescing into plaques in the bilateral inframammary region with incomplete involvement of the folds were observed (Fig 1). A punch biopsy of the area showed degeneration and fragmentation of elastic fibers (Fig 2). Verhoeff – Van Gieson (VVG) staining confirmed the near absence of elastic fibers within the papillary dermis (Fig 3).

**Question 1: What is the diagnosis?**

A. Pseudoxanthoma elasticum (PXE)
B. Pseudoxanthoma-like papillary dermal elastolysis (PXE-PDE)
C. Papillary dermal elastosis (PDE)
D. White fibrous papulosis (WFP)
E. Mid-dermal elastolysis (MDE)

**Answers:**

A. Pseudoxanthoma elasticum – Incorrect. Papillary dermal elastosis (PXE) resembles PXE-PDE, but onsets at a younger age with ocular and cardiovascular manifestations.^1^ On histology with the von Kossa stain, PXE illustrates fragmentation and calcification of elastic fibers.^1^
B. Pseudoxanthoma-like papillary dermal elastolysis – Correct. This is a rare, benign elastic tissue disorder, mainly in women with a clinical similarity to PXE without systemic manifestations.^1^ It presents as asymptomatic white-to-yellow nonfollicular papules forming plaques commonly located in sun-exposed areas like the neck.^1^ Verhoeff – Van Gieson or orcein stains illustrate a total or partial loss of elastic fibers in the papillary dermis in a linear band formation, without calcification, unlike PXE.^2^
C. Papillary dermal elastosis – Incorrect. This appears like PXE-PDE with asymptomatic papules on the neck and upper back, but histology of PDE demonstrates foci of clumped elastic fibers in the papillary dermis with normal reticular dermis.^2^
D. White fibrous papulosis – Incorrect. WFP is similar to PXE-PDE but distinguished from it due to equal presence in both sexes with nonconfluent paler papules, usually on the nape of the neck, lacking the cobblestone appearance.^2^ Histology shows elimination of the papillary dermal elastic plexus with the upper reticular dermis illustrating fibrosis not seen in PXE-PDE.^2^
E. Mid-dermal elastolysis – Incorrect. This is another rare elastolytic disorder presenting with 3 distinct variations: well-demarcated patches of wrinkles on the trunk and proximal extremities (type I); follicular papules on the lateral neck (type II); and reticular erythema with wrinkling.^2^ Histology illustrates paucity of elastic fibers in the mid-dermis, with occasional lymphohistiocytic infiltrate and elastophagocytosis.^2^

**Question 2: Which of the following is NOT a proposed etiologic theory for the development of PXE-PDE?**

A. Ultraviolet (UV) radiation
B. Genetics
C. Aging process
D. Abnormal elastin synthesis
E. Environmental exposure

**Answers:**

A. UV damage – Incorrect. UV exposure is one of the proposed etiologic theories of PXE-PDE. However, UV exposure as the main cause of PXE-PDE is debated as involvement of the axillae, and inframammary folds as in our patient, can occur. In patients with increased melanophages in the dermis, a marker of UV damage in chronic heliodermatitis, the UV radiation etiologic theory may play a role in PXE-PDE development.^2^
B. Genetics – Incorrect. Although less likely, a genetic cause of PXE-PDE is supported by one report of a familial case of PXE-PDE.^3^
C. Aging process – Incorrect. The thinning of oxytalan and elastin within the papillary dermis associated with intrinsic aging has also been postulated as a cause of PXE-PDE and why it occurs in middle-aged individuals.^2^ However, this does not explain why PXE-PDE occurs largely in women and not both sexes.
D. Abnormal elastin synthesis – Incorrect. The dysfunctional elastin synthesis theory has the most supportive evidence with studies using immunohistochemical staining revealing immature elastic fibers in the mid-dermis with a defect in elastin rather than fibrillin-1.^2^ This is further supported by case reports of PXE-PDE in patients on high-dose or prolonged steroid treatment for rheumatologic conditions, illustrating the effect of glucocorticoids on elastin gene expression and messenger ribonucleic acid.^2^
E. Environmental exposure – Correct. Although UV damage is a proposed theory for the development of PXE-PDE, other environmental exposures have not been elucidated as contributing to this disease process.

**Question 3: Which of the following is NOT a treatment option?**

A. Topical retinoids
B. Fraxel laser
C. Clear and brilliant laser
D. Oral retinoids
E. No treatment

**Answers:**

A. Topical retinoids – Incorrect. Most treatments for PXE-PDE are ineffective, however there have been case reports in which topical retinoids have had minimal to marked change with desirable results.
B. Fraxel laser – Incorrect. Fraxel is a nonablative fractional resurfacing laser that has shown up to a 50% improved appearance of the skin after 3 sessions in 1 case report.^4^
C. Clear and brilliant laser – Incorrect. This is also a nonablative fractional resurfacing laser that may be used to treat the cosmetic concerns of PXE-PDE.
D. Oral retinoids – Correct. To date, oral retinoids have not been utilized in the literature to treat PXE-PDE. Additionally, because PXE-PDE is a benign condition, the risks of adverse effects from oral retinoids outweigh the benefits of any potential improvement.
E. No treatment – Incorrect. Once PXE is ruled out, the disease remains benign, and treatment is not necessary unless cosmetic concern exists.

## Conflicts of interest

None disclosed.
